# A Study on the Decay Model of Multi-Block Taxi Travel Demand under the Influence of Major Urban Public Health Events

**DOI:** 10.3390/ijerph19063631

**Published:** 2022-03-18

**Authors:** Feiyi Luo, Zhengfeng Huang, Pengjun Zheng

**Affiliations:** 1 Faculty of Maritime and Transportation, Ningbo University, Ningbo 315211, China; luofeiyi01@163.com (F.L.); zhengpengjun@nbu.edu.cn (P.Z.); 2 Jiangsu Province Collaborative Innovation Center for Modern Urban Traffic Technologies, Nanjing 210096, China

**Keywords:** major public health event, travel demand decay, DIIM, clustering method

## Abstract

A sudden major public health event is likely to have a negative impact on public transport travel for residents, with public travel modes such as the metro and conventional buses experiencing varying degrees of decline in patronage. As a complement to public transport, taxi travel will suffer the same impact. Land use and population density among various functional blocks in a city are different, and therefore their changing rates in taxi travel demand are varied. This paper reveals the taxi travel demand correlations between urban blocks and then constructs a taxi travel demand decay model based on the Dynamic Input-Output Inoperability Model (DIIM) to simulate the decay degree of taxi travel demand in each block. When a major public health event occurs, the residential panic levels in different functional blocks may vary. It results in variable changing speeds of residential travel demand in each block. Based on this assumption, we use the intensity of travel demand as a correlation strength factor between blocks, and equate it with the technical coefficient in the DIIM model. We also define other variables to serve in model construction. These variables include the decay degree of travel demand intensity, residential travel willingness, coefficient of travel demand decay, derivative coefficient of travel demand interdependency, and demand perturbation coefficient. Lastly, we select a central area of Ningbo as the study area, and use taxi travel data in Ningbo during the COVID-19 pandemic of 2020 as input, simulate taxi travel demand dynamics, and analyze the accuracy and sensitivity of the model parameters. The relative errors between the five types of blocks and the actual decay of travel demand intensity are 8.3%, 3.8%, 8.7%, 5.5%, and 5.3%, respectively, which can basically match the actual situation, proving the validity of the model. The results of the study reveal the pattern of taxi travel demand decay among various blocks after major public health events. It provides methodological reference for decision makers to understand the development trend of multi-block taxi travel demand, so as to help form effective emergency plans for different blocks.

## 1. Introduction

Major public health events occur frequently, including infectious atypical pneumonia SARS, avian influenza, influenza A (H1N1), Ebola virus, and novel coronavirus. Much attention is given internationally to analyzing the impact of major public health events on various industries, especially those that need to recruit customers, such as shopping malls, restaurants, and tourism. It is well known there is a very close relationship between travel demand and the boom-and-bust of these industries. If we could know in advance the dynamics of travel demand as a result of the event, it would be useful to estimate the social and economic losses, and to plan further for the resumption of production and the introduction of economic revitalization measures. However, most of these studies provide macrolevel findings on changes of travel demand in larger regions [1,2] and less mention their changes in intracity block division, especially in residential, office building, commercial centers, and transport hubs. It is not conducive to detailed impact findings and recovery guidance measures by zone.

When a major public health and safety event occurs, it has an impact on the normal work and life of the region, and people are less likely to go out due to fear and for reducing the risk of infection. The travel demands of urban residents are constrained by many factors [3] and so present greater vulnerability, especially as residents often commute to work, to and from transport hub areas, public offices, and other places via taxis, and these travel destinations are highly susceptible to disease transmission. In the early stage of a major urban public health event, the hypertransmissibility of the disease and the imperceptible mode of transmission, combined with the introduction of policies such as work stoppages and travel restrictions, inevitably contribute to a reduction in demand for residential taxi trips. However, the travel distribution of taxis will vary from region to region and from time to time. Although the overall demand for residential taxi trips declines after the incident, the demand at special locations such as transport hubs may not always show a precipitous decline. Some of these places even may see a short period of rebound in demand for taxi trips due to factors such as sudden increases in travel out of town. Hospitals and other medical locations are also likely to show different changes in taxi demand compared to other locations. Therefore, it is necessary to analyze the decay pattern of taxi travel demand by block, so as to provide relevant departments with block-by-block data support and help in the development of emergency response plans in the event of a major public health event of the same magnitude in the future.

To analyze the changing extent of travel demand in terms of block level, it is generally necessary to monitor residential dynamic travel trajectory. As to major public health events 10 years ago, there has been less analysis of dynamic travel demand at the block level due to the difficulty of data collection. In the last decade, with the popularity of mobile terminal GPS, we could easily track the individual travel trajectories with mobile phone GPS data, taxi GPS data, and public transport boarding records, which are conducive to exploring the travel demand patterns of different mode groups, and analyzing the daily change in travel demand at the block level. However, analysis of the current daily travel demand is mostly in the context of conventional emergencies, such as special weather, while the analysis of scenarios for major public health events is lacking. Fortunately, a large number of taxi travel data were collected during the COVID-19 pandemic of 2020. These data can be helpful in the calibration of a decay model for taxi travel demand.

In this study, a Dynamic Input-Output Inoperability Model (DIIM) is constructed to implement the multi-block taxi travel demand decay process. The model treats *n* types of blocks in the residential travel system as *n* sectors of the original model, and considers the demand correlation between different blocks to describe the dynamic change process of the travel demand intensity, which can be used to simulate the multi-block travel demand decay law. Subsequently, the calibrated model can be used to provide data support to the relevant authorities in the future when a major public health event of the same magnitude occurs, as well as to assist in the development of emergency response plans. When a similar public health event occurs, the data can be input into the simulation model to quickly obtain results on the changing trend of taxi travel demand between urban blocks.

### 1.1. Review of the Literature

Many scholars have studied issues related to taxi travel demand by blocks. They have mainly focused on various aspects such as spatial and temporal distribution [4], discrimination of functional block and travel hotspot area [5,6], and travel demand forecasting [7]. These studies have carried out block delineation work.

In studies of block delineation based on taxi GPS data, the criteria vary. Cheng et al. [7] divided the blocks of Beijing by using urban roads as partition lines, took the divided blocks as research units, clustered the blocks according to the temporal data of taxi trip occurrence, and then analyzed the temporal and spatial distribution characteristics of passenger trips according to the clustering results. Wang [8] selected five representative areas of different land use functions: office, commercial, transportation hub, around schools, and around hospitals, and then analyzed the temporal change characteristics of taxi travel demand in these five blocks. Chen et al. [9] used a 500 m rule grid as the unit for temporal data extraction of residents’ taxi trips, and performed block clustering to identify the functional characteristics of different areas. Yang [10] established an urban functional area identification model based on XGBoost and LightGBM, and used the Didi taxi data to demonstrate its validation in application. Qi et al. [11] extracted different travel patterns by analyzing the number of pick-ups and drop-offs obtained from the taxi GPS data, and found the pattern was related to the social function of the corresponding land. Pan et al. [12] verified that the social function of urban areas can be identified by the spatial and temporal characteristics of the pick-ups and drop-offs by using a large number of taxi data, and then used real-time taxi data to predict the social function of the test area, and also analyzed the dynamics of social functions in the study area.

The taxi data used in these studies were mostly not related to major special events. A few scholars have also studied taxi travel demand from a city-wide or regional perspective subject to extreme weather (rainfall, snow, high temperature, etc.), epidemics, etc. They mainly carried out studies on travel demand forecasting under these conditions. Liu [13] studied the demand for residents’ taxi trips during rainfall periods by extracting the trajectory data. Farber et al. [14] and Chung et al. [15] analyzed taxi supply and demand on sunny days, rainy days, and snowy days. Kang et al. [16] constructed a multivariate linear regression model to analyze the relationship between different meteorological elements and taxi demand, and finally analyzed the taxi demand changes affected by rainfall in different functional areas. Some scholars have studied the impact of residents’ demand for taxi trips during the epidemic [17,18]. However, these studies did not make predictions when the same situation occurred again in the future. Chang et al. [19] mined the taxi supply and demand matching situation in different regions based on historical taxi data, and, in addition, predicted the taxi travel demand by including weather condition factors.

These studies related to taxi demand under special events have not considered the correlation between the individual blocks and do not mention the analysis of travel demand changes in each block. Many models of system performance degradation under uncertain perturbations can consider the correlation between associated elements. The proposed models include graph-based models, agent simulation models, system dynamics models, IIM models, and DIIM models. Leonardo et al. [20,21,22] developed a graph-based model based on the relationships among the system interior nodes, and the link association between the nodes was described by means of probabilistic analysis. Brown et al. [23] combined a system dynamics model and agent model to study the infrastructure system, and performed a simulation analysis of the performance changes in the system subject to external disturbance. Regarding the process of IIM and DIIM, it is related to Leontief’s theory. Leontief proposed the Leontief economic input–output model in 1951 [24], and later proposed its dynamic style to reflect performance changes for system, which has been widely used [25,26,27] in the field of economy, energy, population issues, environmental protection, human resources, etc. Haimes et al. [28,29] proposed the Input–Output Inoperability Model (IIM) for infrastructure system. Its theory basis comes from Leontief economic input–output model. Crowther et al. [30] proposed a demand-driven IIM model and it could be used to simulate the degree of functional degradation of infrastructure in the event of a disaster. Lian et al. [31] extended the demand-driven IIM model to express system dynamics, the proposed dynamic IIM could be used to analyze the dynamics of interrelated systems under the influence of disasters and attacks. 

Among these methods, IIM and DIIM are widely used in many fields. They aim to capture, inside a ‘simple’ framework, the overall consequences that a negative event may produce in an interdependent scenario. The model analyses how inoperability, that is, incapacity, can correctly perform its own task, how one block influences the other and how inoperability is propagated and amplified due to interdependencies. Many authors use it in the infrastructure failure issue. We could also find some application in the transportation field. For instance, Pant et al. [32] adopted IIM to describe interdependent adverse effects of disruptive events on interregional commodity flows resulting from disruptions at an inland port terminal. Tan et al. [33] used IIM to quantify the impact of disruptions across the entire supply chain. In addition, the DIIM model is more convenient to use, having fewer parameters compared to other models, when modeling the dynamic changes of various components within the system. This method, however, is less applied in the field of transportation. The reason is that the relation between each transportation block is difficult to construct. However, taxi travel data provide an opportunity to construct this matrix.

### 1.2. Research Framework

**Figure 1:**
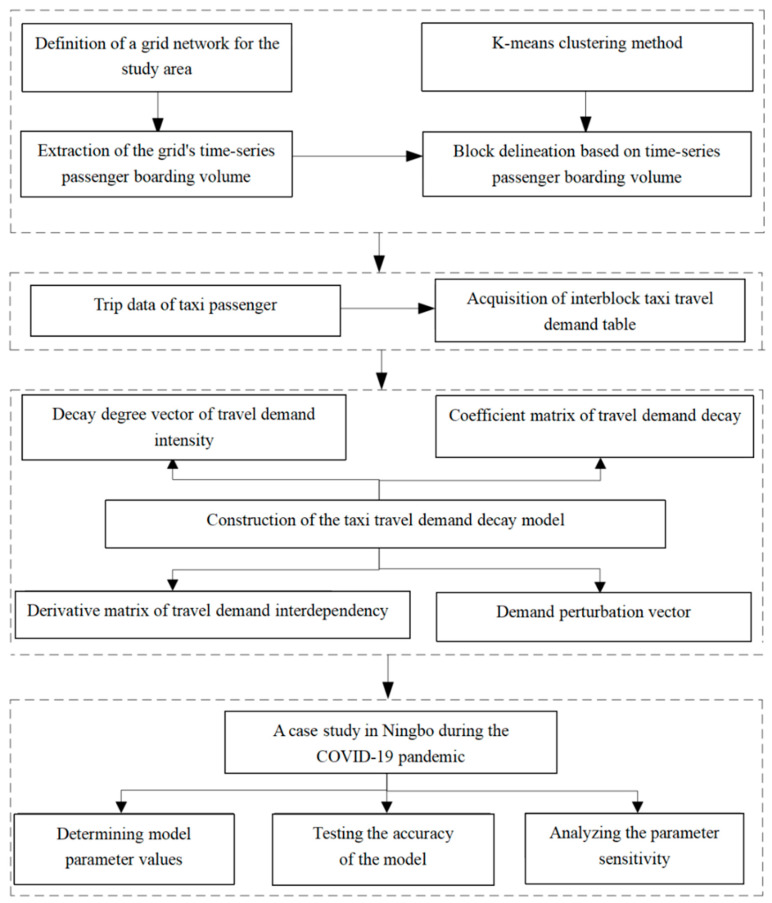
Content of structure framework.

The structural framework for the content is shown in Figure 1. Firstly, the area studied is divided into regular grids, and the temporal boarding passenger volume of each grid is extracted based on taxi GPS data. The *k*-means algorithm is used to cluster the grids based on the temporal passenger boarding volume, and the grids with the same travel characteristics are clustered into one block. Secondly, since all types of blocks are interrelated in terms of travel demand and comprise the taxi travel system, we create the taxi travel demand table between different types of blocks. Because the block correlation influence on travel demand changing procedure at the early stage of a major public health event in the city, we simulate a travel demand intensity decay procedure of the taxi travel system based on the DIIM model. Lastly, we can observe the decay of travel demand intensity for various types of blocks in the system when they are disturbed by major public health events, and also analyze the accuracy and sensitivity of the model.

## 2. Taxi Travel Demand Decay Simulation Based on the DIIM Model

### 2.1. Block Delineation Based on the k-Means Clustering Method

The first step is standardization of the regular grid and passenger boarding volume. Using the grid to divide the studied urban area can help to provide a more refined work. This work can effectively reduce errors when the grid is subsequently clustered into different blocks. When generating the grid, our rule is to ensure that its size is neither too large nor too small. If the grid size is too large, the areas with different land use functions would be combined in the same grid. This result is not conducive to analyzing different characteristics of taxi travel demand. If the grid size is too small, fewer taxi trips are allocated to each grid. It leads to a large number of grids with blank or too few data. In addition, it would also lead to a result that the same building may be divided into different grids. In this research, a 500 m × 500 m grid is used to divide the urban area with reference to other studies, such as Huang et al. [34]. In order to describe the model conveniently, let the number of all grids after cutting be N and the set of passenger boarding volume in each gird be $g\;=\;\left\{g_{1},g_{2},\cdots ,g_{N}\right\}$, where the record of consecutive T days in the ith grid is presented as $g_{i}\;=\;\left\{g_{i1},g_{i2},\cdots ,g_{iT}\right\}$. The multi-day taxi trip $g_{i}$ should be normalized before used as feature value for clustering. In this research, the normalization process is performed by dividing $g_{i}$ by the boarding volume of first day. The normalization formula of multi-day taxi trip for the ith grid follows:

(1) $$g_{i}^{\prime }\;=\;\left\{1,\frac{g_{i2}}{g_{i1}},\cdots ,\frac{g_{iT}}{g_{i1}}\right\}$$

The feature value set of all grid taxi trips after standardization is presented as Ω; the formula follows:

(2) $$\Omega \;=\;\left\{g_{i}^{\prime }|g_{i}^{\prime }=\left(g_{i1}^{\prime },g_{i2}^{\prime },\cdots ,g_{iT}^{\prime }\right),i=1,2,\cdots ,N\right\}$$

The next step is block clustering. Based on the normalized grid time-varying volumes, *k*-means clustering is carried out to acquire blocks with similar feature values. These different types of blocks are necessary to build the decay model. Because each block has similar changes in taxi trips during the time period studied, it could act as a products sector in traditional IIM when a major urban public health event occurs. The follow-up considers residential taxi trips in a travel system, in which different types of blocks play as aforementioned product sectors.

### 2.2. Acquisition of Interblock Taxi Travel Demand Table

In the early stage of a major public health event, taxi trips vary in each block. It makes the travel demand between blocks change significantly. A table of taxi travel demand between different types of blocks is shown below. In this table, the total demand, interblock travel demand, external attraction, and external production could act as the amount produced, intermediate demand, final demand, and total of value added in traditional IIM, respectively. 

In Table 1, $x_{ij}$ represents the number of taxi passengers that boarded at block i and were dropped off at block j. $C_{i}$ represents the taxi demand that its destination is an external block and the origin is block i. $X_{i}$ represents all taxi trips generated by block i. $Z_{j}$ denotes the taxi demand generated in an external block whose destination is block j. In Table 1, there are two types of equilibrium. The horizontal balance can be expressed as $\sum_{j=1}^{n}x_{ij}\;+\;C_{i}\;=\;X_{i}$; the vertical balance can be expressed as $\sum_{i=1}^{n}x_{ij}\;+\;Z_{j}\;=\;X_{j}$.

### 2.3. Construction of the Taxi Travel Demand Decay Model

#### 2.3.1. IIM Model

Based on economist Wassily Leontief’s work on economic interdependencies, the IIM models the effects of a perturbation in a network through an expression of interdependency throughout the network. Rather than using economic impacts, the IIM looks to analyze the propagation of “risk of inoperability”. The risk of inoperability, or simply inoperability, is the key adaptation to Leontief’s I–O model. Inoperability could be defined as “the inability for a system to perform its intended function”. Inoperability is likened to the concept of “unreliability”, which describes the probability of failure for a system. Inoperability is expressed as a value between 0 and 1 where 0 denotes no inoperability (flawless operation) and 1 denotes complete inoperability (complete failure).

In view of the correlation of residents’ taxi trips between different types of blocks, these blocks are considered to be the components of residents’ taxi trip system. The production and attraction volume of taxi passengers are viewed as an “input” or “output” of a block. The matrix notation of the IIM for resident’s taxi trip system is presented as follows:

(3) $$q\;=\;A^{*}q\;+\;C^{*}$$

In Formula (3), $A^{*}$ is the interdependency matrix (*n* × *n* matrix) derived from the taxi travel demand table (the explanation of $A^{*}$ is in Formula (5)). $C^{*}$ is the demand disturbance vector of the travel system. It could be represented as the demand change between the inside and outside of the system when the system is subject to a major urban public health event (the explanation of $C^{*}$ is in Formula (6)). Vector q is a collection of inoperability values for all *n* blocks modeled in the travel system. This vector indicates the level of lost taxi travel trips of all blocks. This trip loss is generated by some perturbation related to the major public health event. When the assessed value is 0, it corresponds to the state of the system under normal conditions. When the assessed value is 1, it corresponds to a complete breakdown of the system with no travel volume. 

To better understand vector q, we use the following physical form to show it. $\overset{⌢}{X}$ represents the vector of normal resident taxi trips and $\overset{⌣}{X}$ represents the vector of remaining resident taxi trips after the travel system was disturbed by a major urban health event:

(4) $$q\;=\;\left[{\left(diag\left(\overset{⌢}{X}\right)\right)}^{-1}\left(\overset{⌢}{X}-\overset{⌣}{X}\right)\right]$$

We could use Formula (5) to express $A^{*}$, where $A\;=\;{\left(a_{ij}\right)}_{n\times n}\;=\;{\left(x_{ij}/X_{j}\right)}_{n\times n}$ is a direct consumption coefficient matrix. The element $a_{ij}$ represents the ratio of taxi travel demand of block direction *i* → *j* to the total number of attraction demand in block j:

(5) $$A^{*}\;=\;\left[{\left(diag\left(\overset{⌢}{X}\right)\right)}^{-1}A\left(diag\left(\overset{⌢}{X}\right)\right)\right]$$

$C^{*}$ can be expressed as the following equation. In Formula (6), $\overset{⌢}{C}$ represents the demand attraction vector from inside to outside blocks when the travel system is at the normal level; $\overset{⌣}{C}$ represents the demand attraction vector from inside to outside blocks when the travel system is perturbed by a major urban public health event:

(6) $$C^{*}\;=\;\left[{\left(diag\left(\overset{⌢}{X}\right)\right)}^{-1}\left(\overset{⌢}{C}-\overset{⌣}{C}\right)\right]$$

#### 2.3.2. DIIM Model

An assumption in IIM is that the disturbance coefficients are constant, implying that the strength of disturbance is fixed before, during, and after a crisis. Such an assumption is very unrealistic since it is expected that the disturbance will change, especially in major catastrophic events. The classic IIM is unable to manage these dynamic dependencies. To overcome this limitation, in what follows, a suitable extension of the IIM methodology is provided. According to the Leontief dynamic input–output model, the demand dynamics in the residential taxi travel system are modeled as follows:

(7) $$X\left(t\right)\;=\;AX\left(t\right)\;+\;B\dot{X}\left(t\right)\;+\;\tilde{C}\left(t\right)$$

The vector X(t) represents taxi trips generated by different types of blocks on the *t*th day. The vector $\tilde{C}\left(t\right)$ represents the attracted demand of external blocks on the *t*th day. The matrix *B* is a square *n* × *n* matrix that represents the willingness of the residents to travel in the emergency situation. A diagonal matrix *B* in the form $B\;=\;-K^{-1}$ substitutes into Formula (7), and we can obtain the following equation:

(8) $$\frac{d\left(X\left(t\right)\right)}{d\left(t\right)}\;=\;-K\left[X\left(t\right)-AX\left(t\right)-\tilde{C}\left(t\right)\right]$$

When *t* = 0, we name the corresponding Formula (8) as the initial formula. We use a separate subtraction operation for each side of the initial formula and Formula (8), and multiply each side by ${\left(diag\left(\overset{⌢}{X}\right)\right)}^{-1}$; we can then obtain:

(9) $$\dot{q}\left(t\right)\;=\;K\left[A^{*}q\left(t\right)+C^{*}\left(t\right)-q\left(t\right)\right]$$

It is more useful to consider a discrete time representation of the DIIM. An approximation for the derivative $\dot{q}\left(t\right)$ is given by q(t+1) − q(t), and then the discrete-time DIIM is given by:

(10) $$q\left(t+1\right)\;=\;K\left[A^{*}q\left(t\right)+C^{*}\left(t\right)-q\left(t\right)\right]\;+\;q\left(t\right)$$

The equation above finds the inoperability or decay values on the (*t* + 1)th day. $C^{*}\left(t\right)$ is the disturbance vector of taxi travel demand on the *t*th day. 

When a major public health event occurs, the intensity of travel demand in the taxi travel system is gradually declining. In order to present a situation consistent with the change in travel demand intensity, we could use the following remaining demand level Q(t) to replace q(t):

(11) Q(t) = 1−q(t)

### 2.4. Determination of Model Parameters

**Figure 2:**
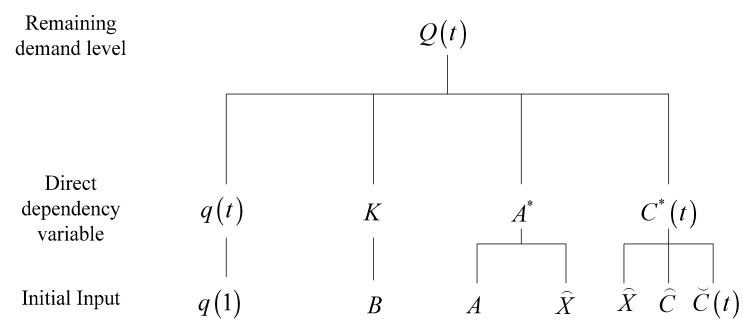
Parameter diagram.

The required variable Q(t) in the taxi travel demand decay model relates to the following parameters: q(t), decay degree vector of travel demand intensity; K, coefficient matrix of travel demand decay; and $A^{*}$, derivative matrix of travel demand interdependency; $C^{*}\left(t\right)$, demand disturbance vector. The relationship among the parameters involved in the model is shown in Figure 2.

#### 2.4.1. Decay Degree Vector of Travel Demand Intensity

As to these *n* blocks in the residential travel system of the *t*th day, the decay degree of travel demand intensity is expressed as $q\left(t\right)\;=\;{\left[q_{1}\left(t\right),q_{2}\left(t\right),\cdots ,q_{n}\left(t\right)\right]}^{T}$. Without the major public health event, the entire travel system is in a stable state. This stable state can be used as the initial input of demand decay degree on the first day. Stated differently, we assign $q\left(1\right)\;=\;{\left[0,0,\cdots ,0\right]}^{T}$. That is to say, the taxi demand intensity in the initial travel system is $Q\left(1\right)\;=\;1\;-\;q\left(1\right)\;=\;{\left[1,1,\cdots ,1\right]}^{T}$.

#### 2.4.2. Coefficient Matrix of Travel Demand Decay

In this study, the decay coefficient matrix is strongly correlated with the residential travel intention matrix, expressed as K = −1/B. Matrix *B* is the residential travel intention matrix, represented as $B\;=\;diag\left(B_{1},B_{2},\cdots ,B_{n}\right)$. When no special events occur, let B = 0, indicating that the travel demand intensity of the entire system is in steady state and residents have a general travel pace. When a block is affected by a negative special event, such as experiencing the epidemic spread of the new coronavirus and reaching a certain level of panic, residential travel willingness would be rapidly decreasing. In this situation we could let its value be less than 0. When there is a positive special event, such as holding a major event, travel willingness would be strong and we set it to be larger than 0. For convenience, we set the matrix of residential travel willingness B = diag(−1,−1,⋯,−1) in our research and obtain the decay coefficient matrix K = diag(1,1,⋯,1).

#### 2.4.3. Derivative Matrix of Travel Demand Interdependency

Equation (5) is used to solve the demand derivative matrix $A^{*}$. Its value relates to the demand dependency direct consumption coefficient matrix A and the travel demand volume vector $\overset{⌢}{X}\;=\;{\left[{\overset{⌢}{X}}_{1},{\overset{⌢}{X}}_{2},\cdots ,{\overset{⌢}{X}}_{n}\right]}^{T}$. 

#### 2.4.4. Demand Perturbation Vector

The demand disturbance vector is expressed as $C^{*}\left(t\right)\;=\;{\left[C_{1}^{*}\left(t\right),C_{2}^{*}\left(t\right),\cdots ,C_{n}^{*}\left(t\right)\right]}^{T}\;=\;\left[{\left(diag\left(\overset{⌢}{X}\right)\right)}^{-1}\left(\overset{⌢}{C}-\overset{⌣}{C}\left(t\right)\right)\right]$. It is related to the initial travel demand $\overset{⌢}{C}\;=\;{\left[{\overset{⌢}{C}}_{1},{\overset{⌢}{C}}_{2},\cdots ,{\overset{⌢}{C}}_{n}\right]}^{T}$ between each internal block and the outside block, the initial travel volume $\overset{⌢}{X}\;=\;{\left[{\overset{⌢}{X}}_{1},{\overset{⌢}{X}}_{2},\cdots ,{\overset{⌢}{X}}_{n}\right]}^{T}$ of each block, the travel demand $\overset{⌣}{C}\left(t\right)\;=\;{\left[{\overset{⌣}{C}}_{1}\left(t\right),{\overset{⌣}{C}}_{2}\left(t\right),\cdots ,{\overset{⌣}{C}}_{n}\left(t\right)\right]}^{T}$ between each internal block, and the outside block on the *t*th day after the occurrence of a major public health event. $C_{i}^{*}$ takes on values ranging from 0 to 1. We assume that $C^{*}\left(t\right)$ follows a curve function. We use different parameters to calibrate the curve to make it match the corresponding disturbance scenario.

## 3. Case Studies

### 3.1. Overview of the Study Area

**Figure 3:**
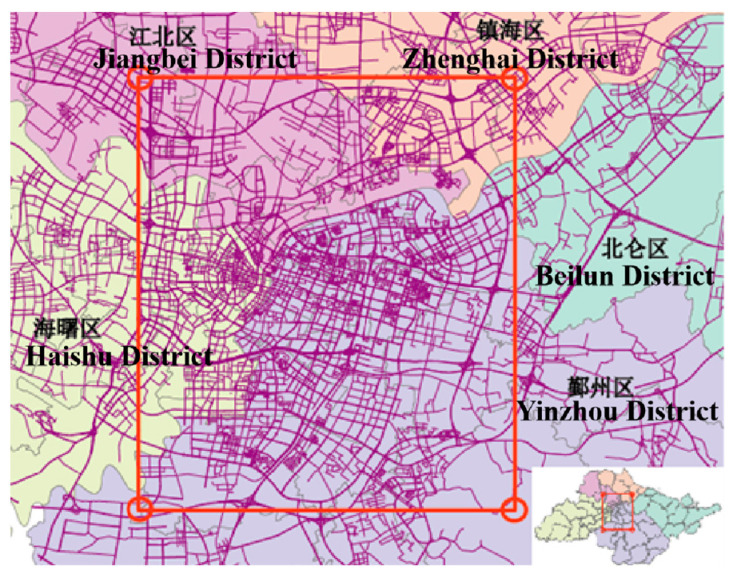
Study area.

Ningbo’s land area is 9816 sq km, and the urbanization zone covers a total area of 453 sq km. Owing to the large area, this paper does not study the whole city, but selects the more densely populated zone as the study area, which contains five districts: Jiangbei, Zhenhai, Haishu, Yinzhou, and Beilun. Its range is in the latitude from 121.505° N to 121.665° N and longitude from 29.784° E to 29.947° E, as shown in Figure 3. There are important transportation hubs, office buildings, commercial streets, and residential zones in the study area. Because this area selected is in the center of Ningbo City, its road network is densely distributed and residents travel more frequently herein than other areas. Supporting data are sufficient to study the taxi travel demand in this area.

### 3.2. Data Pre-Processing

Ningbo City on 22 January 2020 issued an emergency notice to citizens about the new coronavirus and set up a leading group for the prevention and control of the new coronavirus epidemic. On 23 January 2020, Zhejiang Province initiated a first-level public health emergency response, and thus Ningbo City closed various public places and suspended various large events and religious activities. There was a sudden decrease in the number of taxi trips on these days. Therefore, we selected the taxi GPS data in Ningbo from 22 January 2020 to 10 February 2020, which was the initial period of a major public health event. The taxi GPS information includes pick-up location (latitude and longitude) and time stamp along with drop-off location and time stamp. 

**Figure 4:**
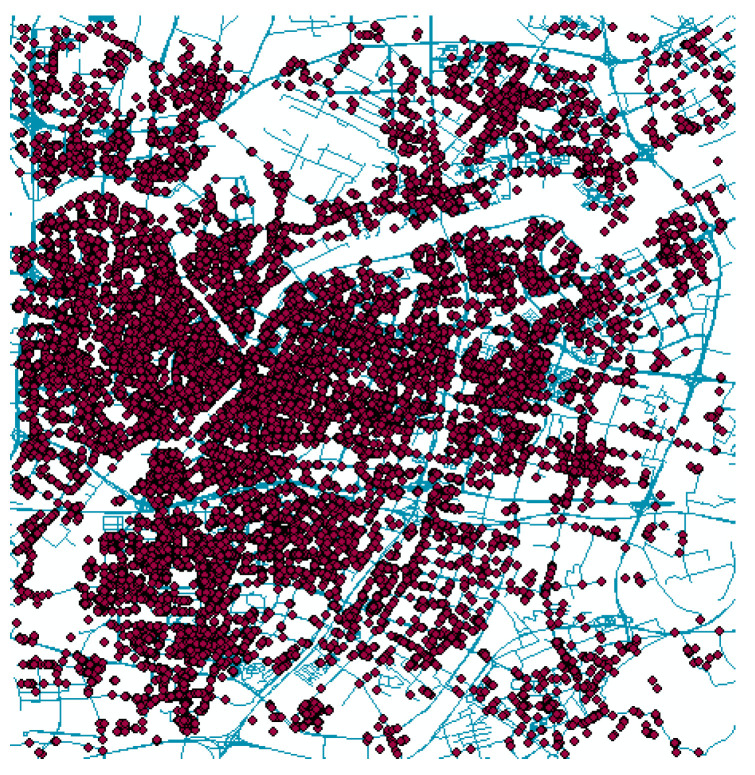
Taxi GPS points of passenger boarding.

Taking the data of 22 January 2020 as an example, the passenger boarding points plotted using ArcMap is shown in Figure 4. Through the processing of some unreasonable data, the remaining valid taxi GPS data are about 454,500 in total.

### 3.3. Block Classification Based on k-Means Clustering

The study area was finally divided into 1116 grids by selecting a regular grid of 500 m × 500 m for block classification. Taking two consecutive days as a time interval, there are 10 time intervals for analysis. If we analyze the features of grids by the passenger boarding number per unit time, we find that the grids with no taxi trip records account for 8.2%, mostly in areas of underdeveloped places, park green land, etc. The grids without taxi trip records for 1–9 unit times account for 35.2%, and their taxi trips per unit time is very low, less than four trips per grid. In order to select grids with more data to analyze, we keep the remaining 56.6% grids. These grids average 35.13 taxi trips per unit time per grid. These relevant statistics are shown in Table 2.

**Figure 5:**
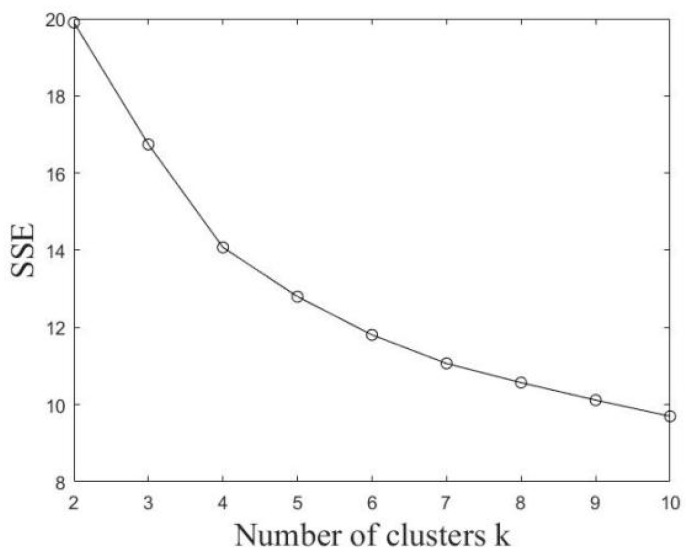
SSE value changes with *k* value.

**Figure 6:**
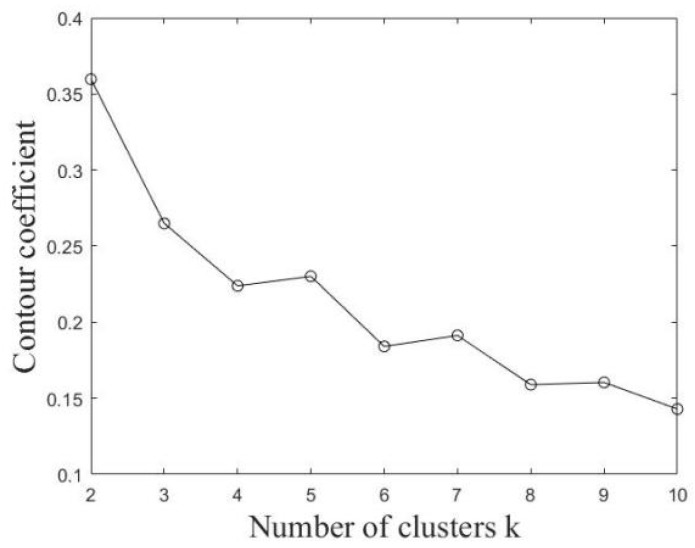
Contour coefficient changes with *k* value.

Based on the time-series taxi trips extracted from the selected grids, *k*-means is used to cluster the grids. In order to determine the optimal cluster number k, two indicators, the sum of squares due to error (SSE) and contour coefficient, are used. The variation of these indicators with the change in the number of clusters is shown in Figure 5 and Figure 6. If SSE is smaller and the contour coefficient is larger, it indicates a better clustering effect. According to their changing trend, the number of clusters was comprehensively determined to be five.

**Figure 7:**
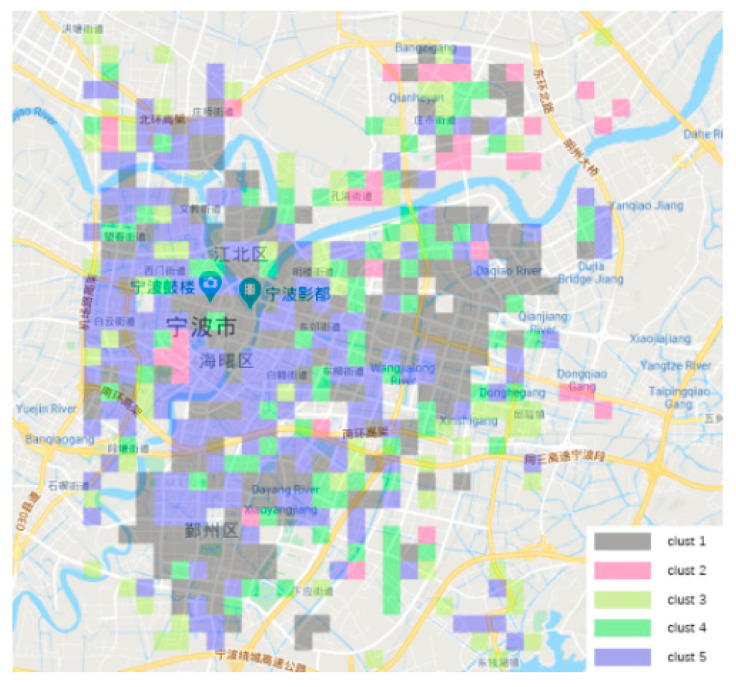
Land distribution of five blocks.

**Figure 8:**
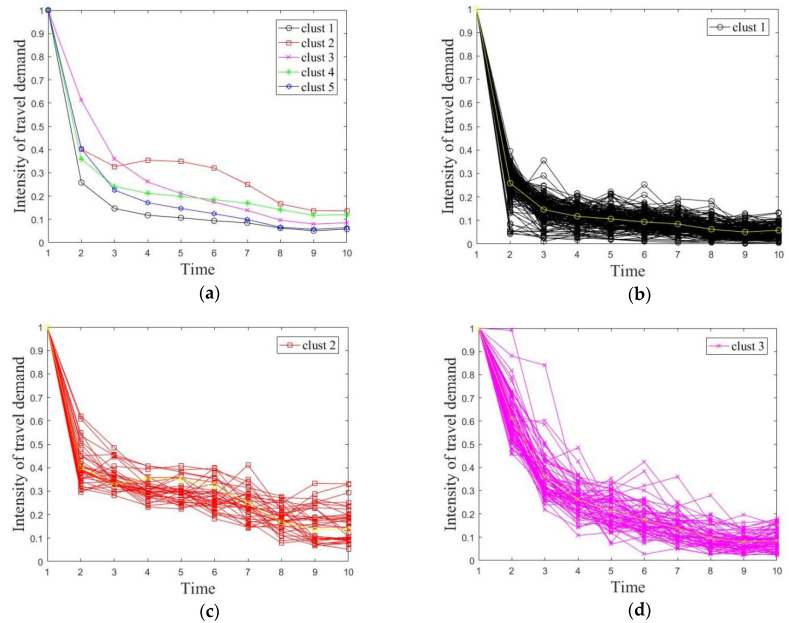

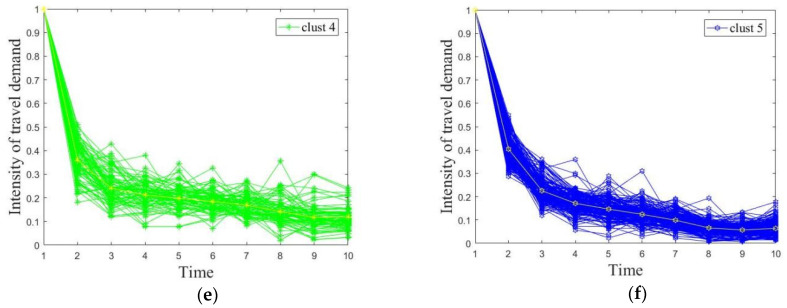
Time-series taxi trips of each block. (**a**) Time-series taxi trips of all blocks. (**b**) Time-series taxi trips of block #1. (**c**) Time-series taxi trips of block #2. (**d**) Time-series taxi trips of block #3. (**e**) Time-series taxi trips of block #4. (**f**) Time-series taxi trips of block #5.

By clustering the valid grids to form five blocks, the acquired land distribution and time-series taxi trips of each block are shown in Figure 7 and Figure 8. We can describe different features of these five blocks as follows: (1) Clust1 is mostly an employment area, mainly distributed in the southern business district and consisting of office buildings and public service areas. It was greatly affected by the shutdown policy, so the initial decline in taxi travel demand intensity was the fastest among the five categories. (2) Clust2 is mostly a transportation hub area, located near railway stations and highway bus stations. Its taxi travel demand intensity did not keep declining similar the other blocks, but the demand intensity rebounded in the middle phase, reflecting the decision change by some residents from waiting at previous position to departing from the city. (3) Clust3 is mostly a suburban residential area with poor transportation accessibility and less rail transit and bus coverage. This type of land has less taxi travel demand even in the normal situation, and thus the taxi travel demand intensity declined at the slowest rate compared to other types of land in the emergency period. (4) Clust4 is mostly educational and medical areas, including Ningbo No. 2 Hospital, the Higher Education Park, etc. The taxi travel demand intensity showed a sharp drop in the beginning, followed by a more stable trend. One of the reasons for the stable trend may be that some patients would go to hospitals for physical examinations. (5) Clust5 is mostly residential areas with dense road networks, and the overall trend of taxi travel demand intensity was similar to that of the first category. Its changing trend was slightly weaker than the first category.

### 3.4. Travel Demand Table and Other Parameters

Table 3 shows the balance of residential taxi travel demand between the five types of blocks. The largest demand occurs inside the first block. Travel demand is less within the third block. The strongest demand connection is between type 1 and 5 of the blocks. The lowest demand connection is between type 2 and 3 of the blocks.

Based on the taxi demand between the five types of blocks, the relative demand intensity between the various types of blocks can be obtained as follows and expressed as matrix A.

(12) $$A\;=\;\left(\begin{array}{ccccc} 0.358 & 0.229 & 0.318 & 0.325 & 0.345\\ 0.055 & 0.036 & 0.074 & 0.068 & 0.065\\ 0.043 & 0.043 & 0.045 & 0.049 & 0.040\\ 0.102 & 0.090 & 0.112 & 0.107 & 0.100\\ 0.299 & 0.236 & 0.254 & 0.278 & 0.298 \end{array}\right)$$

Since the travel vector for each type of block in the normal case is $\overset{⌢}{X}\;=\;{\left[\begin{array}{ccccc} 43196 & 10313 & 5907 & 13547 & 37363 \end{array}\right]}^{T}$, the associated derivative matrix $A^{*}$ can be calculated by Equation (5) as:

(13) $$A^{*}\;=\;\left(\begin{array}{ccccc} 0.358 & 0.055 & 0.044 & 0.102 & 0.298\\ 0.230 & 0.036 & 0.042 & 0.089 & 0.236\\ 0.314 & 0.075 & 0.045 & 0.112 & 0.253\\ 0.325 & 0.069 & 0.049 & 0.107 & 0.276\\ 0.346 & 0.065 & 0.040 & 0.101 & 0.298 \end{array}\right)$$

Regarding the demand disturbance vector $C^{*}\left(t\right)$, we calibrate their function distribution through fitting. It was found that all the blocks except for the second type conform to a power function distribution. The land use of the second block mostly relates to transportation hub areas, whose demand disturbances are more in line with a polynomial distribution. This function makes it more suitable to explain the traffic return at railway stations and bus stations at a certain time. The final demand disturbance vector $C^{*}\left(t\right)$ of all the blocks is expressed as follows.

(14) $$C_{1}^{*}\left(t\right)\;=\;0.587t^{-2.117}\;+\;0.155$$

(15) $$C_{2}^{*}\left(t\right)\;=\;-0.006t^{3}\;+\;0.102t^{2}\;-\;0.551t\;+\;1.028$$

(16) $$C_{3}^{*}\left(t\right)\;=\;0.233t^{-6.239}\;+\;0.155$$

(17) $$C_{4}^{*}\left(t\right)\;=\;0.532t^{-2.321}\;+\;0.109$$

(18) $$C_{5}^{*}\left(t\right)\;=\;0.440t^{-2.988}\;+\;0.155$$

### 3.5. Analysis of Model Simulation Results

**Figure 9:**
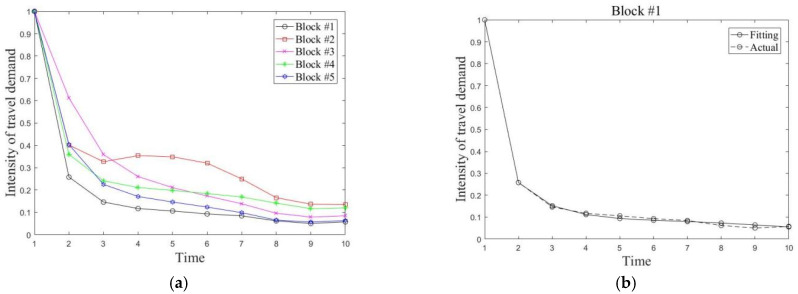

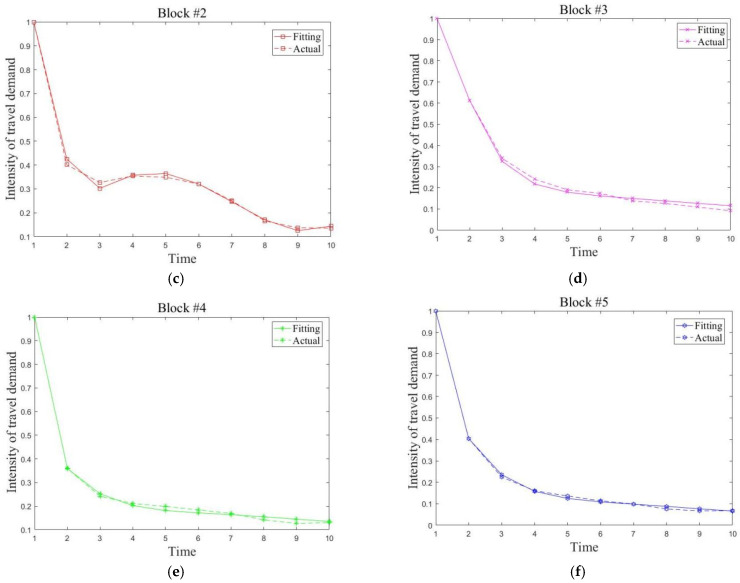
Demand intensity trend acquired by simulation. (**a**) Demand intensity simulation for all blocks. (**b**) Demand intensity simulation for block #1. (**c**) Demand intensity simulation for block #2. (**d**) Demand intensity simulation for block #3. (**e**) Demand intensity simulation for block #4. (**f**) Demand intensity simulation for block #5.

The simulation results of demand intensity for the five different types of blocks are acquired by the taxi travel demand decay model, and are shown in Figure 9a. The graph reflects the change of travel demand intensity over time for each type of block when a major public health event occurs. The simulated travel demand intensity for the five types of blocks is basically in line with the actual situation. The average relative errors between the simulation results and the actual values for the five types of blocks are 8.3%, 3.8%, 8.7%, 5.5%, and 5.3%, respectively. The simulation error is slightly larger for the third category of blocks because of a rebound in travel volume in the mid-term, but it still meets the requirement.

### 3.6. Sensitivity Analysis

The impact of severity level on the simulation result is analyzed by adjusting the parameters in the demand perturbation vector $C^{*}\left(t\right)$ for each type of block. As the trend of travel demand intensity for the second block is special, we do not conduct a detailed analysis for its perturbation change. Specifically, we conduct sensitivity analysis under the perturbation influence by other blocks, i.e., adjusting $c_{i2}$ in the power function distribution $C_{i}^{*}\left(t\right)\;=\;c_{i1}t^{c_{i2}}\;+\;c_{i3},\;i\;=\;1,3,4,5$ to obtain the influence on the trend of travel demand intensity for each type of block. As varied blocks are interrelated, changes in the parameter of one block also have an effect on the other blocks. To better describe the overall impact of parameter changes on these five types of blocks, the fine-tuned changing values of the parameters are set to (−0.2, −0.1, +0.1, +0.2), and an indicator w is introduced to indicate the change in system average performance.

**Figure 10:**
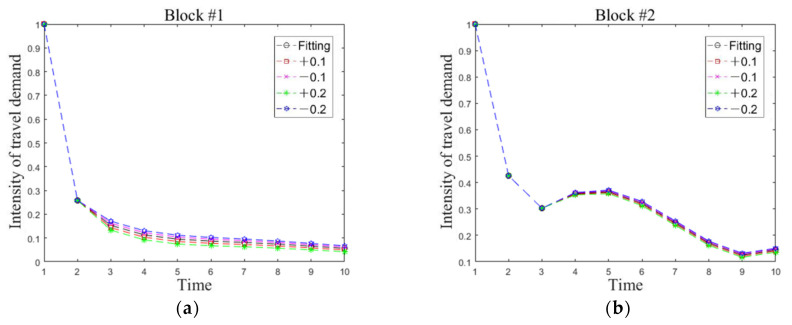

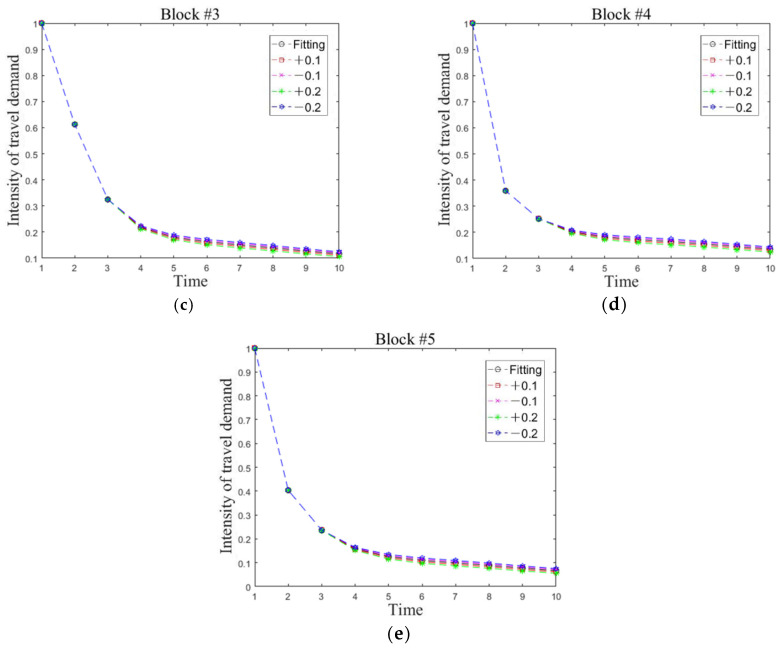
The impact of changes in parameter *c*_12_ on the demands of all blocks. (**a**) Demand sensitivity of block #1. (**b**) Demand sensitivity of block #2. (**c**) Demand sensitivity of block #3. (**d**) Demand sensitivity of block #4. (**e**) Demand sensitivity of block #5.

Table 4 and Figure 10 show the sensitivity change results. Almost each type of block responds with high sensitivity to changes in parameter $c_{12}$. One of the reasons is that the first block belongs to workplace, which is a land use with strong demand attraction. The first block is more closely linked to other blocks in terms of the relative demand intensity; therefore, the simulation results are most sensitive to changes in $c_{12}$. Observing the graphs, we find a slight change in $c_{12}$ would have the greatest impact on the first and fifth blocks, since there is a strong demand association between these blocks. In addition, as time evolves, it has a pronounced separation between simulation result and reality, i.e., the parameter changes have less impact on the separation in the former phase and more on the later phase.

By observing the simulation results caused by the adjustment of perturbation parameters in other blocks, we can establish the following conclusions: (1) The change of parameter $c_{32}$ has a relatively small impact on all types of blocks. (2) The change of parameter $c_{42}$ or $c_{52}$ has a great impact on their own blocks and the first block. In other words, except for the suburban residential block, changes in the parameters of all other blocks have a significant impact on the demand simulation results of the first block. It implies that the employment-based block has the greatest demand sensitivity to major public health events. 

## 4. Discussion

Urban taxi travel demands rely on taxi trip generation of all the blocks in the city. Owing to the relevance of each block, if one of the blocks is in lockdown, it would have a serious impact on the taxi travel demand of citizens. In the current city, each block has become highly dependent and interdependent, each one relying (directly or indirectly) on the taxi travel demands provided by the others. Owing to the presence of interdependencies, a failure in any block may easily propagate to the others, with the result of affecting a large, unpredictable set of travelers.

This study shed insights into the modeling of interblock taxi travel demand decay. It is modeled with the DIIM, which is a method of analysis that captures cascading effects of a disturbance in interdependent travel systems. It is unique for several reasons. First, a scarcity of studies investigated the impact of interblock travel demand under major urban public health events, no mention of taxi travel demand. Second, few studies focused on changing travel demand features of varied blocks during these events, which entails many collected data. Although different approaches have been proposed to estimate possible impacts of block travel demand under the COVID-19 pandemic, they did not consider interdependence among blocks. Furthermore, most are mainly devoted to identifying the travel features of the whole city or a certain block. For one instance, Zafri et al. [35] conducted a survey during the COVID-19 pandemic. Their study finds that 56% and 45% of the respondents were expected to increase travel by walking and cycling, respectively. They were reluctant to travel by public transport, such as a taxi. For another instance, as restrictions tightened during the COVID-19 pandemic, Haddawy et al. [36] recorded a greater decrease in the number of long trips compared to short trips in rural border area. They reported a decrease in short trips of 32% and a decrease in long trips of 70%. As to taxis, few studies could be found that focused on their travel demand during the COVID-19 pandemic. Actually, since the appearance of ride-hailing services such as Didi in China, the number of vacant taxis is far greater than before, even in regions with high-density road networks and developed commercial centers (Huang et al.) [34]. The occurrence of the COVID-19 pandemic would make it worse. Thus, we should care about the changing features of taxi travel during this special period. In our study, we found that two blocks would undergo the greatest taxi travel demand change. We could also simulate this changing trend. Furthermore, we analyzed the taxi travel demand sensitivity when the disturbance changed. These analyses have rarely been studied in other research.

In addition, the following key contribution is highlighted. The interdependency A* matrix derives from the taxi travel demand table, which considers the strength of connected blocks in the travel system. This simplification leads to a general model and increases the potential for its use.

Regarding the application, this study is devoted to decision makers, able to discover elements neglected by the experts. It could help the analysts to identify the most critical blocks, that is, the elements which need to be more (and urgently) protected. Specifically, it results in the ability of traffic managers to recognize the effect of decay across an entire travel network in the city. 

The current work is focused mainly on calibration of parameters to make the model better simulate the interblock travel demand decay. Although the current calibration could meet our case requirement, it would change according to other scenarios. The C* decay vector requires probabilistic failure distributions and decay curves that have not been readily collected for every scenario. Future work will concern more application scenarios to collect different rules for the C* decay vector. If these kinds of data are provided, we could also match the event and taxi travel demand changing rule. This matching work could help to detect anomalies according to taxi data, such as indicated in the work of Davis et al. [37] and Belhadi et al. [38]. Moreover, in some situations the degree of dependency depends also on the operational conditions of the travel system. Future work will concern how to introduce more factors into the interdependency A* matrix construction to make the model closer to the actual situation. These factors include weather, travel condition, and policy.

Although this study mainly focuses on the dynamic impact of public health events on taxi travel demand, we also consider the relationship between public health environment and taxi travel demand. By tracking the travel data during the normalization (the year 2020–2021) of the coronavirus epidemic, we found that the relation between public health environment and taxi travel demand changed. As long as people’s public health knowledge and psychological capacity are improved, public health events will not have an extreme impact on taxi travel demand. When coronavirus mutation variants appeared occasionally during the years 2021–2022, mostly the corresponding disease occurred in confined spaces such as food markets and entertainment clubs. They were rarely spread inside the taxis. With the help of experts in the analysis of diseases and their preventive measures, the environment of public health has been improved. People have effectively protected themselves by actively wearing masks when entering public places. It reduced the risk of airborne transmission of diseases. In addition, the transportation administration has urged taxi companies to adopt effective disinfection measures inside the vehicles; they used propaganda to make people believe they are safe in the vehicles. Under the combined effect of these factors, people are no longer afraid of choosing public travel modes such as taxis. As a result, the impact of the normalized epidemic on taxi travel demand has been reduced. In the future, we will use the travel data for the years 2021–2022 to further analyze the impact of varied levels of accumulated knowledge in corresponding public health infectious disease on taxi travel demand.

## 5. Conclusions

In order to explore the changing demand for taxi trips in different blocks during the initial stage of major urban public health events, so as to provide a basis for relevant authorities to formulate corresponding measures to reduce social and economic losses, we establish a taxi demand decay model based on DIIM. We treat taxi trips in different blocks as components of a taxi trip system and consider the correlation of taxi travel demand between different types of blocks. The model could analyze the dynamic changes in the intensity of taxi travel demand between different types of blocks within a city at the beginning of a major public health event. The tasks accomplished and the conclusions obtained are as follows.

We divided the city of Ningbo into five categories of blocks (employment, transport hub, suburb residential, educational and medical, and downtown residential) through time-series taxi demand clustering. Based on the statistical taxi travel demand table between the five blocks, the taxi travel demand intensity is obtained and it is used for DIIM model construction. The model application shows the relative errors of demand trend between the simulated and the actual situations, which were 8.3%, 3.8%, 8.7%, 5.5%, and 5.3%, respectively. They were relatively small, indicating that the model established is feasible and effective. The sensitivity analysis reveals that the people in the employment-based blocks were more sensitive to the external disturbance, while suburban residents were the least sensitive.

The future work includes mining data under different public health event scenarios to test the stability of the model. In addition, we will take into account more factors such as weather, travel condition, and policy influence when modeling. This simulation model will then be closer to the actual situation.

## Figures and Tables

**Table 1:** Taxi travel demand table between different types of blocks.

Blocks	1	2	…	*j*	…	*n*	External Attraction	Total Demand
1	$x_{11}$	$x_{12}$	…	$x_{1j}$	…	$x_{1n}$	$C_{1}$	$X_{1}$
2	$x_{21}$	$x_{22}$	…	$x_{2j}$	…	$x_{2n}$	$C_{2}$	$X_{2}$
…	…	…	…	…	…	…	…	…
i	$x_{i1}$	$x_{i2}$	…	$x_{ij}$	…	$x_{in}$	$C_{i}$	$X_{i}$
…	…	…	…	…	…	…	…	…
n	$x_{n1}$	$x_{n2}$	…	$x_{nj}$	…	$x_{nn}$	$C_{n}$	$X_{n}$
External production	$Z_{1}$	$Z_{2}$	…	$Z_{j}$	…	$Z_{n}$	——	——
Total demand	$X_{1}$	$X_{2}$	…	$X_{j}$	…	$X_{n}$	——	——

**Table 2:** Number distribution of grids in terms of the varied taxi trip feature.

Total Time Intervals without Trips	0	1	2	3	4	5	6	7	8	9	10
Number of grids	632	66	58	41	42	31	39	31	38	47	91
Percentage (%)	56.6	5.9	5.2	3.7	3.8	2.8	3.5	2.8	3.4	4.2	8.2
Average daily taxi trips	35.13	3.43	2.25	1.59	1.07	0.67	0.48	0.25	0.16	0.07	0.00

**Table 3:** Taxi travel demand balance table for various types of blocks.

Different Types of Blocks	Type 1	Type 2	Type 3	Type 4	Type 5	External Block	Amount of Demand
Type 1	15,481	2360	1878	4399	12,904	6175	43,196
Type 2	2360	368	439	925	2429	3793	10,313
Type 3	1878	439	266	659	1500	1166	5907
Type 4	4399	925	659	1448	3754	2363	13,547
Type 5	12,904	2429	1500	3754	11,116	5661	37,363

**Table 4:** Sensitivity analysis table.

Variables	Changing Values	Demand Sensitivity of the Various Types of Blocks (%)					System Average Performance Change w (%)
		Type 1	Type 2	Type 3	Type 4	Type 5	
$c_{12}$	+0.1	−7.99	−1.31	−2.30	−2.19	−3.88	−3.53
	−0.1	7.05	1.16	2.05	1.95	3.46	3.13
	+0.2	−17.08	−2.78	−4.89	−4.64	−8.25	−7.53
	−0.2	13.29	2.20	3.89	3.70	6.54	5.92
$c_{32}$	+0.1	−0.01	0	−0.01	0	−0.01	−0.01
	−0.1	0.01	0	0.01	0	0	0
	+0.2	−0.01	0	−0.02	−0.01	−0.01	−0.01
	−0.2	0.01	0	0.02	0.01	0.01	0.01
$c_{42}$	+0.1	−1.04	−0.30	−0.50	−1.64	−0.84	−0.86
	−0.1	0.93	0.28	0.47	1.45	0.75	0.78
	+0.2	−2.22	−0.64	−1.12	−3.50	−1.78	−1.85
	−0.2	1.77	0.51	0.89	2.73	1.42	1.46
$c_{52}$	+0.1	−1.21	−0.33	−0.57	−0.56	−1.52	−0.84
	−0.1	1.09	0.30	0.52	0.50	1.36	0.75
	+0.2	−2.55	−0.70	−1.21	−1.17	−3.22	−1.77
	−0.2	2.08	0.57	0.99	0.96	2.58	1.44
